# Supplementary material for: Temporal Eating Patterns and Eating Windows among Adults with Overweight or Obesity
Source: Nutrients. 2021 Dec 15;13(12):4485. doi: 10.3390/nu13124485 (PMC8705992; doi:10.3390/nu13124485)
Supplement: Supplementary file 1 [file nutrients-13-04485-s001.zip › nutrients-1458470-supplementary.pdf]

Supplemental Material

Table S1. Eating patterns by study on adherent days (n=84).

Variable	*TEP (n=35)*	*TREAT (n=49)*	*p-value*
Eating Occasions (#/day)	3.9 [3.4-4.7]	4.0 [3.1-4.5]	0.706
First Eating Occasion, (hh:mm)	9:42 [8:36-10:17]	9:40 [8:54-10:33]	0.511
Last Eating Occasion, (hh:mm)	19:55 [19:13-21:13]	19:34 [19:02-21:02]	0.376
95% Eating Duration, (h, min)	14h 02min [12h 57min-15h 12min]	14h 07min [13h 00min-15h 20min]	0.978
Eating midpoint, (hh:mm)	14:30 [13:36-15:02]	14:13 [13:28-15:11]	0.602
Within-person variability total daily eating occasions, (%CV)	28.0 [21.1-34.3]	30.0 [22.6-39.2]	0.257
Within-person variability First Eating Occasion, (h, min)	18.3 [13.6-23.1]	20.7 [15.1-27.6]	0.259
Within-person variability Last Eating Occasion, (h,min)	8.6 [6.5-13.3]	9.4 [7.5-11.5]	0.824

TEP, temporal eating patterns; TREAT, time-restricted eating; %CV, percent coefficient of variation; significance *p<0.05. Eating occasions were not normally distributed and therefore reported as median [interquartile range]. Eating pattern analyzes were generated off eating events that included adherent days (> 2 EO logged >5 h apart). First and last eating occasion, and eating midpoint are reported as local times (hh:mm).

Table S2. Eating patterns and within-person variability from all eating occasions (n=84).

Variable	All (n=84)	Male (n=23)	Female (n=61)	p-value Male v Female	Weekday	Weekend	p-value weekday v weekend
Eating Occasions (#/day)	3.7 [2.5 -4.4]	3.7 [2.8-4.4]	3.7 [2.5-4.4]	0.946	3.7 [2.4 -4.3]	3.0 [2.3-4.3]	0.063
First Eating Occasion, (hh:mm)	10:21 [09:03-11:10]	10:24 [08:57-11:27]	10:21 [09:16-11:03]	0.711	10:13 [08:59-11:10]	10:15 [09:09-11:07]	0.46
Last Eating Occasion, (hh:mm)	18:54 [17:45-19:59]	18:57 [18:27-20:48]	18:52 [17:37-19:39	0.188	18:48 [17:39-20:00]	19:13 [16:49-20:59]	0.792
95% Eating Window, (h, min)	14h 09min [12h 57min-15h 16min]	14h 08min [12h 40min-15h 26min	14h 09min [12h 57min-15h 04min	0.992	13h 49min [12h 39min - 15h 09min]	11h 51min [10h 08min-13h 32min]	<0.0001*
Eating midpoint, (hh:mm)	14:26 [13:24-15:16	14:28 [14:12 -15:19]	14:13 [13:22-15:05]	0.189	14:32 [13:21-15:17]	14:06 [12:45-15:40]	0.622
Within-person variability eating occasion frequency, (%CV)	38.0 [28.0-46.0]	31.5 [23.8-41.2]	40.0 [31.0-47.0]	**0.023**	36.0 [28.0-46.0]	33.0 [20.0-43.0]	0.15
Within-person variability First Eating	5h 53min [4h 17min-8h 05min]	5h 11min [4h 13min- 7h 55min]	5h 58min [4h 18min-8h 05min]	0.452	5h 48min [4h 09min-8h 19min]	2h 58min [1h 27min-5h 24min]	<0.0001*

Occasion, (h, min)							
Within-person variability Last Eating Occasion, (h, min)	4h 03min [2h 39min-5h 31min]	3h 12min [2h 24min- 4h 33min]	4h 14min [2h 44min-5h 44min]	0.114	3h 48min [2h 06min-5h 38min]	2h 44min [1h 10min-4h 34min]	0.171

%CV, percent coefficient of variation; significance *p<0.05. EOs were not normally distributed and therefore reported as median [interquartile range]. First and last eating occasions, as well as eating midpoint are reported as local times (hh:mm). Aggregate data from the TEP and NY-TREAT studies were included.

Table S3. Non-Parametric correlations between eating pattern variables and anthropometrics (n=85).

	**Body weight (kg)**	**BMI (kg/m^2)**	**WC (cm)**	**HC (cm)**	**WHR**
Eating Occasions (#/day)	r= 0.098<br>p=0.374	r=0.125<br>p= 0.256	r=0.164<br>p= 0.142	r= 0.102<br>p= 0.360	r= 0.068<br>p= 0.541
First Eating Occasion (hr:mm)	r= -0.194<br>p= 0.077	r= -0.157<br>p= 0.155	r= -0.122<br>p= 0.274	r= -0.055<br>p= 0.623	r= -0.054<br>p= 0.630
Last Eating Occasion (hr:mm)	r= -0.083<br>p= 0.453	r= -0.096<br>p= 0.386	r= 0.042<br>p= 0.711	r= -0.001<br>p= 0.990	r= 0.052<br>p= 0.642
95% Eating Window (h, min)	r= -0.010<br>p= 0.923	r= -0.076<br>p= 0.490	r= 0.065<br>p= 0.564	r= -0.007<br>p= 0.950	r=0.063<br>p= 0.572
Eating Midpoint (hr:mm)	r= -0.161<br>p= 0.145	r= -0.115<br>p= 0.297	r= 0.029<br>p= 0.799	r= -0.031<br>p= 0.783	r= 0.109<br>p= 0.329

BMI, body mass index; WC, waist circumference; HC, hip circumference; WHR, waist-to-hip ratio; Pearson correlations for normally distributed variables and Spearman for non-normally distributed variables; significance *p<0.05.

Table S4. Correlations between eating pattern variables and glycemic outcomes in the TEP study (n=35).

	**HbA1c (%)**	**Fasting Glucose (mg/dL)**	**Fasting Insulin (mIU/L)**	**HOMA-β**	**HOMA-IR**
Eating Occasions (#/day)	r= -0.117<br>p= 0.504	r= -0.069<br>p= 0.695	r= 0.295<br>p= 0.085	r= 0.297<br>p= 0.018*	r= 0.277<br>p= 0.107
First Eating Occasion (hh:mm)	r= -0.280<br>p= 0.104	r= -0.077<br>p= 0.660	r= 0.076<br>p= 0.664	r= 0.021<br>p= 0.905	r= 0.079<br>p= 0.651
Last Eating Occasion (hh:mm)	r= -0.193<br>p= 0.266	r= 0.122<br>p= 0.484	r= 0.305<br>p= 0.075	r= 0.176<br>p= 0.309	r= 0.322<br>p= 0.059
95% Eating Window (h, min)	r= 0.122<br>p= 0.487	r= 0.068<br>p= 0.698	r= 0.114<br>p= 0.516	r= 0.130<br>p= 0.455	r= 0.116<br>p= 0.508
Eating Midpoint (hr:mm)	r= -0.077<br>p= 0.660	r= -0.076<br>p= 0.663	r= 0.291<br>p= 0.090	r= 0.240<br>p= 0.165	r= 0.230<br>p= 0.081

HbA1c, glycated hemoglobin; HOMA-β, homeostasis model assessment for beta-cell function; HOMA-IR, homeostasis model assessment for insulin resistance; significance *p<0.05; Pearson correlations for normally distributed variables and Spearman for non-normally distributed variables

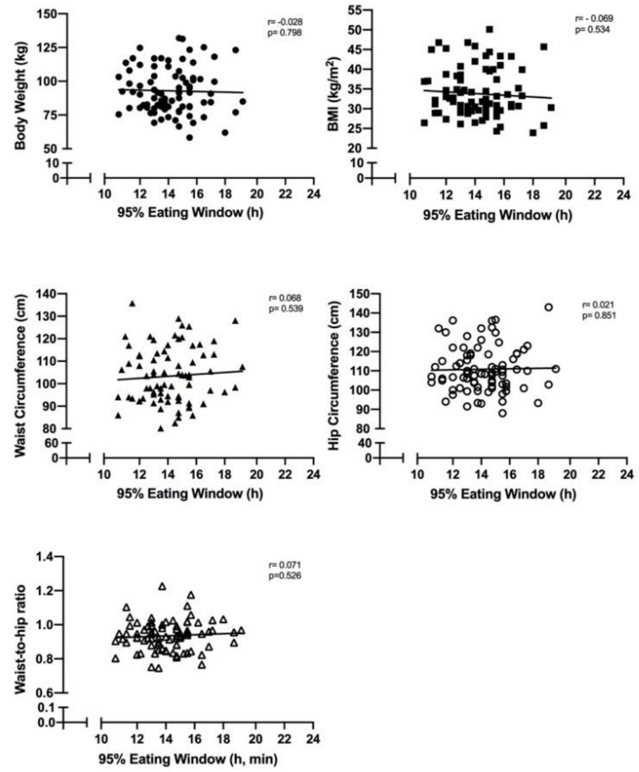

Figure S1. Associations Between the 95% Eating Window and Anthropometrics in Adults with Overweight and Obesity. There was no significant difference between the 95% eating window and body weight, BMI, waist and hip circumference and waist-to-hip ratio. p<0.05; significance in bold.

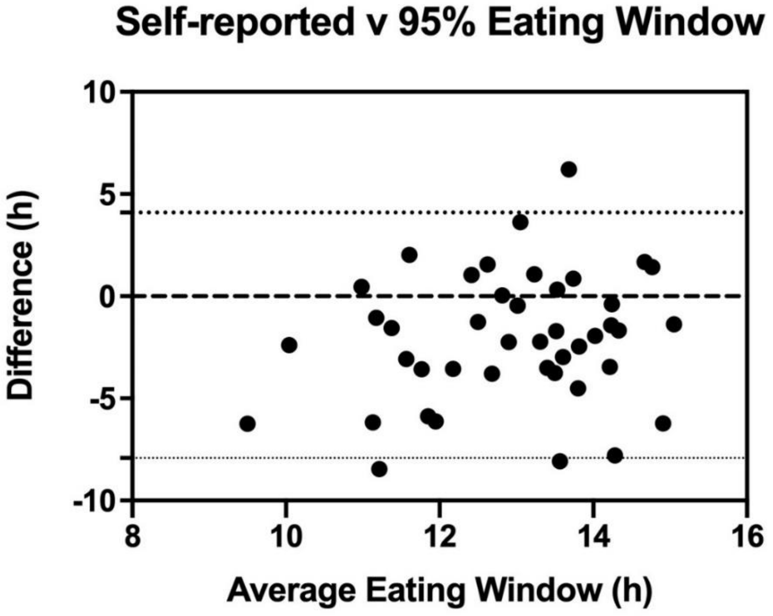

Figure S2. Bland Altman Plot analysis comparing the 95% eating window and self-reported eating window. The mean bias (-1h 50min ± 2h 56min) is represented by a dashed, bolded line and 95% limits of agreement (-7h 36min to 3h 55min) by short dashed lines.
